# Gas6 in chronic liver disease—a novel blood-based biomarker for liver fibrosis

**DOI:** 10.1038/s41420-023-01551-6

**Published:** 2023-08-02

**Authors:** Katharina Staufer, Heidemarie Huber, Jasmin Zessner-Spitzenberg, Rudolf Stauber, Armin Finkenstedt, Heike Bantel, Thomas S. Weiss, Markus Huber, Patrick Starlinger, Thomas Gruenberger, Thomas Reiberger, Susanne Sebens, Gail McIntyre, Ray Tabibiazar, Amato Giaccia, Heinz Zoller, Michael Trauner, Wolfgang Mikulits

**Affiliations:** 1 Department of General Surgery, Division of Transplantation, Medical University of Vienna, Vienna, Austria; 2 Department of Internal Medicine III, Division of Gastroenterology & Hepatology, Medical University of Vienna, Vienna, Austria; 3 Center for Cancer Research, Comprehensive Cancer Center Vienna, Medical University of Vienna, Vienna, Austria; 4 Division of Gastroenterology and Hepatology, Department of Internal Medicine, Medical University of Graz, Graz, Austria; 5 Department of Medicine I, Gastroenterology, Hepatology and Endocrinology, Medical University of Innsbruck, Innsbruck, Austria; 6 Department of Gastroenterology, Hepatology and Endocrinology, Hannover Medical School, Hannover, Germany; 7 Center for Liver Cell Research, Children’s University Hospital (KUNO), University of Regensburg Hospital, Regensburg, Germany; 8 Department of Anesthesiology and Pain Therapy, Inselspital, University Hospital Bern, Bern, Switzerland; 9 Department of Surgery, Division of General Surgery, Medical University of Vienna, Vienna, Austria; 10 Clinicum Favoriten, HPB Center, Vienna Health Network and Sigmund Freud Private University, Vienna, Austria; 11 Christian-Doppler Laboratory for Portal Hypertension and Liver Fibrosis, Medical University of Vienna, Vienna, Austria; 12 Institute for Experimental Cancer Research, Kiel University and University Hospital Schleswig-Holstein (UKSH) Campus Kiel, Kiel, Germany; 13 Aravive Biologics, Houston, TX USA

**Keywords:** Diagnostic markers, Liver fibrosis

## Abstract

The expression of the receptor tyrosine kinase Axl and its cleavage product soluble Axl (sAxl) is increased in liver fibrosis, cirrhosis, and hepatocellular carcinoma (HCC). In this multicenter study, we evaluated the diagnostic value of Gas6, the high-affinity ligand of Axl, in patients with chronic liver disease. Levels of sAxl and Gas6, and their albumin (alb) ratios were analyzed in serum samples of patients with biopsy-proven liver fibrosis, end-stage liver disease, HCC, and healthy controls, and were compared to Fibrosis-4 (FIB-4), enhanced liver fibrosis (ELF™) test, Child-Pugh score (CPS), model of end-stage liver disease (MELD) score, hepatic venous pressure gradient, and α-fetoprotein, respectively. A total of 1111 patients (median age 57.8 y, 67.3% male) was analyzed. Gas6/alb showed high diagnostic accuracy for the detection of significant (≥F2: AUC 0.805) to advanced fibrosis (≥F3: AUC 0.818), and was superior to Fib-4 for the detection of cirrhosis (F4: AUC 0.897 vs. 0.878). In addition, Gas6/alb was highly predictive of liver disease severity (Odds ratios for CPS B/C, MELD ≥ 15, and clinically significant portal hypertension (CSPH) were 16.534, 10.258, and 12.115), and was associated with transplant-free survival (Hazard ratio 1.031). Although Gas6 and Gas6/alb showed high diagnostic accuracy for the detection of HCC in comparison to chronic liver disease patients without cirrhosis (AUC 0.852, 0.868), they failed to discriminate between HCC in cirrhosis versus cirrhosis only. In conclusion, Gas6/alb shows a high accuracy to detect significant to advanced fibrosis and cirrhosis, and predicts severity of liver disease including CSPH.

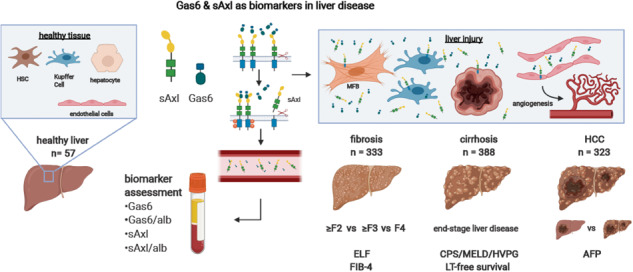

## Introduction

Chronic liver disease (CLD) is one of the leading causes of morbidity and mortality worldwide and constitutes an increasing healthcare burden [1]. The main causes of CLD in Western countries are alcohol-related liver disease (ALD), non-alcoholic fatty liver disease (NAFLD), and viral hepatitis, which may lead to liver fibrosis, cirrhosis, hepatocellular carcinoma (HCC), and liver failure requiring transplantation [2].

Early detection of hepatic fibrosis in patients at risk is crucial for optimal treatment allocation. For diagnosing fibrosis, liver biopsy has remained the gold standard in terms of accuracy [3]. Non-invasive testing by vibration controlled transient elastography (VCTE) or shear-wave elastography has gained popularity in clinical practice as a screening instrument for liver fibrosis [4] but shows substantial variability in patients with ascites and obesity, or may be false positive after food intake or in the situation of liver congestion [5–8]. To overcome obstacles of these diagnostic tools, efforts have led to the development of several blood-based biomarkers for the assessment of fibrosis. These include (i) non-patented tests which can be performed at no to low additional costs, but show less good performance than VCTE or (ii) patented serum markers, which show better performance than unpatented tests but cause higher costs [8].

The non-patented fibrosis-4 (FIB-4) test which includes age, platelets, and liver enzymes (alanine aminotransferase, ALT; aspartate aminotransferase, AST) has been recommended as screening instrument to identify patients with CLD in the out-patient setting, while the patented Enhanced Liver Fibrosis™ (ELF™) test was suggested to be used as an instrument to confirm advanced fibrosis or cirrhosis in patients with intermediate to high risk (i.e., VCTE ≥ 8 kPa) [8]. Furthermore, non-invasive tests, either VCTE or serum-based biomarkers, have some value in the prediction and risk stratification for clinically significant portal hypertension (CSPH), liver decompensation and HCC. However, in the absence of sufficient data, it has remained unclear which patient subpopulations may be safely excluded from invasive assessment of hepatic venous pressure gradient (HVPG) or regular HCC screening [8].

The receptor tyrosine kinase (RTK) Axl, a member of the TAM family (Tyro3, Axl, MerTK), and its high-affinity ligand Gas6 were shown to be implicated in the development of fibrosis [9–11] and HCC [11–13]. Mechanistically, Gas6/Axl signaling promotes fibrosis through HSC hepatic stellate cell (HSC) activation, and upregulation of Axl in HCC fosters tumor progression by affecting HCC plasticity and remodeling the tumor environment [9, 14, 15]. Mice harboring a deletion of Axl or Gas6 show decreased susceptibility to steatosis, steatohepatitis, and fibrosis [9, 16]. In line with a crucial role of Gas6/Axl in liver diseases, we have previously demonstrated that soluble Axl (sAxl), generated by Axl shedding, is elevated in patients with liver fibrosis and cirrhosis, and may detect early stages of HCC [10, 17]. We showed that the accuracy of sAxl to detect significant to advanced fibrosis, and cirrhosis could be further improved by applying a sAxl/albumin ratio (sAxl/alb), making it a suitable screening parameter especially in a situation where VCTE is not available [18]. Yet, the role of Gas6 as serum-based screening marker for the detection of liver fibrosis, compensated and decompensated liver cirrhosis, and HCC has remained unclear.

In the current study, we investigated the performance of Gas6 and Gas6/alb for the detection and prediction of significant to advanced liver fibrosis, compensated and decompensated liver cirrhosis, CSPH, transplant-free survival, and HCC in a large Central European multicentric cohort.

## Results

### Study population

**Fig. 1:**
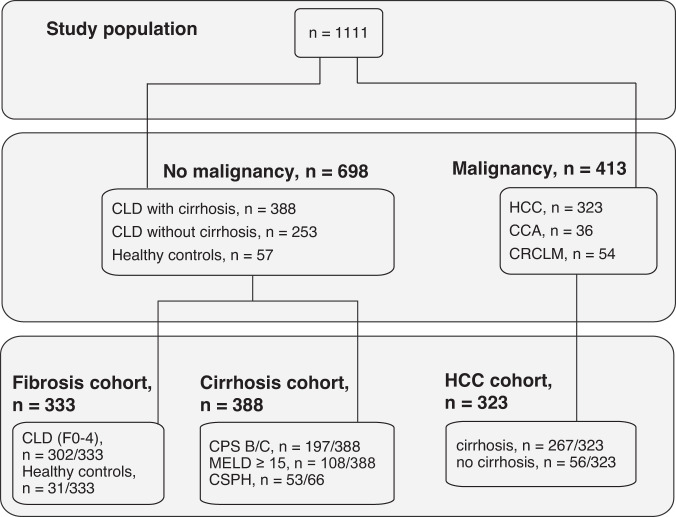
Study population. CCA cholangiocarcinoma, CLD chronic liver disease, CPS Child Pugh stage, CRCLM colorectal carcinoma liver metastases, CSPH clinically significant portal hypertension, HCC hepatocellular carcinoma, MELD model of end-stage liver disease.

A total of 1111 patients (median age 57.8 years (y), 67.3% male) was included into the study (Fig. 1, Supplementary Table S1). Patients were recruited at five university medical centers in Austria and Germany as follows: Medical University of Vienna (*n* = 398), Medical University of Graz (*n* = 176), Medical University of Innsbruck (*n* = 408), Hannover Medical School (n = 38), and University Hospital Regensburg (n = 91). The study population was stratified in a fibrosis cohort (*n* = 333), a cirrhosis cohort (*n* = 388), and an HCC cohort (*n* = 323) (Fig. 1).

### Gas6 levels and Gas6/albumin ratios to detect significant to advanced liver fibrosis

The diagnostic accuracy for the detection of significant to advanced fibrosis was investigated in serum samples of 302 patients with liver disease (biopsy-proven fibrosis grades 0 to 4) and 31 healthy volunteers from two university medical centers in Austria (Medical University of Vienna, *n* = 248; Medical University of Graz, *n* = 85) (Fig. 1). Table 1 displays the patient characteristics of the fibrosis cohort.

**Table 1:** Patient characteristics of patients with chronic liver disease and healthy controls: Fibrosis cohort.

Fibrosis cohort	All patients *N* = 333	Vienna cohort *N* = 248	Graz cohort *N* = 85	*p*-value
Age (y); median (Q1;Q3)	50.8 (37.8;59.4)	50.0 (36.2;59.8)	52.0 (43.0;58.5)	0.148
Male sex; *n* (%)	177 (53.1)	135 (54.4)	42 (49.4)	0.461
BMI (kg/m^2^) median (Q1;Q3)	26.8 (23.8;32.7)	27.4 (24.0;34.8)	26.0 (23.5;28.5)	0.011
Liver Disease, *n* (%)	302 (90.7)	217 (87.5)	85 (100)	
Liver disease etiology, *n* (%)				
NAFLD	150 (45.0)	117 (53.9)	33 (38.8)	
Viral hepatitis	83 (24.9)	46 (21.2)	37 (43.5)	
AIH/PSC/PBC/Overlap	43 (12.9)	31 (14.3)	12 (14.1)	
DILI	8 (2.4)	6 (2.8)	2 (2.4)	
ALD	10 (3.0)	10 (4.6)	0 (0.0)	
Cryptogenic	8 (2.4)	7 (3.2)	1 (1.2)	
Healthy controls	31 (9.3)	31 (12.5)	0 (0.0)	
Liver cirrhosis	61 (18.3)	48 (19.4)	13 (15.3)	0.214
CPS	5 (5;7)	5 (5;8)	5 (5;6)	0.076
MELD	8.7 (7.5;12.1)	8.8 (7.5;14.1)	8.1 (7.5;10.1)	0.354
Non-invasive biomarkers				
sAxl (ng/ml)	45.2 (36.4;60.3)	43.4 (35.6;57.7)	53.0 (42.3;70.1)	0.001
sAxl/alb*10	10.3 (8.1;14.2)	10.0 (7.9;11.7)	12.1 (8.9;11.8)	0.008
Gas6 (ng/ml)	34.4 (27.1;49.3)	33.4 (26.4;48.2)	38.2 (29.9;52.5)	0.025
Gas6/alb*10	7.8 (6.2;11.7)	7.7 (5.9;11.7)	8.6 (6.6;11.8)	0.148
ELF™ (ng/ml), *n* = 277	9.0 (8.3;10.1)	9.2 (8.3;10.6)	8.7 (8.1;9.5)	0.008
Liver biopsy, *n* (%)	307 (92.2)	222 (89.5)	85 (100)	
F0	68 (20.4)	46 (20.7)	22 (25.9)	0.330
F1	82 (24.6)	59 (26.6)	23 (27.1)	0.932
F2	65 (19.5)	49 (22.1)	16 (18.8)	0.533
F3	31 (9.3)	20 (9.0)	11 (12.9)	0.306
F4	61 (18.3)	48 (21.6)	13 (15.3)	0.214

None of the patients had any malignancy or benign liver tumor. In five of 31 healthy subjects, liver disease was ruled out by liver biopsy.

*BMI* body mass index, *NAFLD* non-alcoholic steatohepatitis, *AIH* autoimmune liver disease, *PSC* primary sclerosing cholangitis, *PBC* primary biliary cholangitis, *DILI* drug induced liver disease, *ALD* alcoholic liver disease, *CPS* Child Pugh Score, *MELD* Model of End-stage Liver Disease, *alb* albumin.

**Fig. 2:**
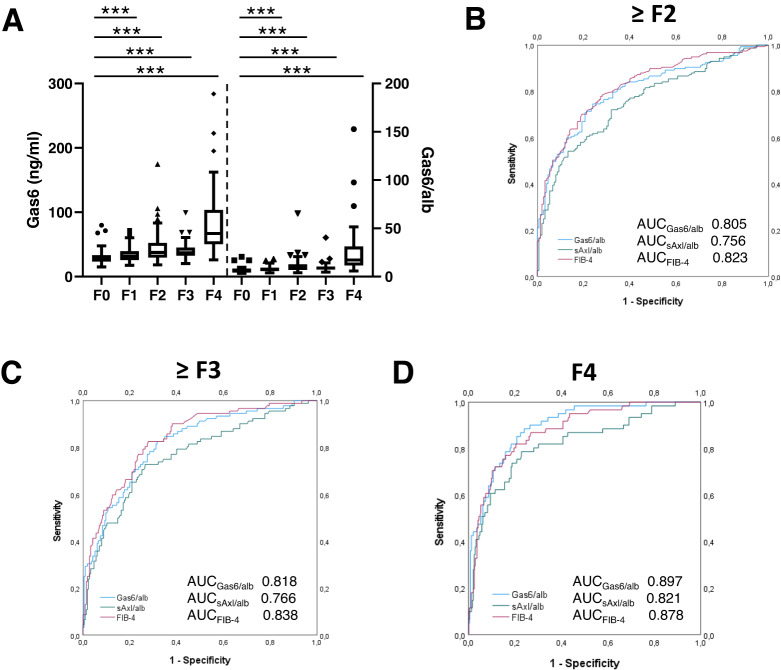
Gas6, Gas6/alb, and sAxl/alb in liver fibrosis. **A** Analysis of Gas6 and Gas6/alb serum levels in patients without fibrosis (F0, *n* = 68), with mild fibrosis (F1, *n* = 82), moderate fibrosis (F2, *n* = 65), advanced fibrosis (F3, *n* = 31) and cirrhosis (F4, *n* = 61) as assessed by liver biopsy. **B**–**D** Comparison of the diagnostic accuracy shown as area under the curve (AUC) of sAxl/alb and Gas6/alb and FIB-4 for **B** significant fibrosis (≥ F2) and **C** advanced fibrosis (≥ F3) as well as (**D**) liver cirrhosis (F4) according to fibrosis grading based on liver histology. Outliers are marked. F, fibrosis grade. Statistical significant differences are expressed as asterisks: ***P < 0.001.

Gas6 levels and the Gas6/alb ratios significantly increased with fibrosis stage (Fig. 2A), similar to sAxl and sAxl/alb as observed previously [10]. However, the diagnostic accuracy of Gas6/alb for F ≥ 2, F ≥ 3, and F4 was superior to that of sAxl, sAxl/alb, and Gas6 (Supplementary Table S2). In addition, Gas6/alb outperformed FIB-4 for the detection of F4 (AUC 0.897 vs. 0.878) (Fig. 2B–D), and was similar to that of the ELF™ test (AUC 0.904) (Supplementary Table S2). When applying a cut-off of 9.61 for Gas6/alb, a sensitivity, specificity, PPV, and NPV of 88.5%, 76.0%, 47.8%, and 96.4% were achieved (Supplementary Table S2). When using a balanced random sub-sampling cross-validation the diagnostic accuracy of sAxl, Gas6, and their albumin ratios was confirmed (Supplementary Table S3).

As shown previously, sAxl and sAxl/alb for fibrosis detection were independent of sex and BMI [18]. In the present study, Gas6 and Gas6/alb levels were independent of BMI, but were significantly lower in male patients. Median (Q1;Q3) Gas6 levels in male and female patients were 32.52 ng/mL (24.91;44.25) and 37.15 ng/ml (29.37;52.54) (*p* < 0.001), while median Gas6/alb levels were 7.03 (5.33;10.03) and 8.35 (Q1;Q3:6.98;12.89) (*p* < 0.001), respectively. If put in a multivariable binary logistic regression model, sex was significantly associated with ≥F3 and F4, but not with ≥F2.

### Gas6 levels and Gas6/albumin ratios to predict decompensated cirrhosis, end-stage liver disease (ESLD), and CSPH

**Fig. 3:**
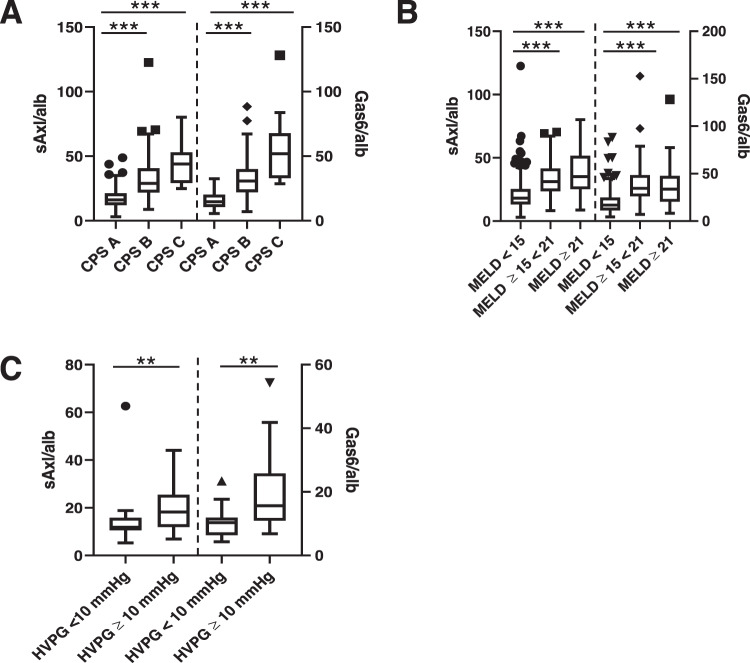
sAxl/alb and Gas6/alb in patients with liver cirrhosis. **A** Analysis of sAxl/alb and Gas6/alb serum levels in cirrhosis patients according to CPS A (*n* = 159), CPS B (*n* = 176), and CPS C (*n* = 279). **B** Analysis of sAxl/alb and Gas6/alb serum levels in cirrhosis patients according to low MELD (< 15, *n* = 280), medium MELD (≥15 and <21, *n* = 106) and high MELD scores (≥21, *n* = 30). **C** sAxl/alb and Gas6/alb serum levels in cirrhosis patients showing a low HVPG (< 10 mm Hg, *n* = 13) and a significantly increased HVPG (≥10 mm Hg, *n* = 59). CPS Child Pugh stage, HVPG hepatic venous pressure gradient, MELD model of end-stage liver disease. Statistical significant differences are expressed as asterisks: **P < 0.01, ***P < 0.001.

The patient cohort consisted of 388 patients of three University Medical Centers in Austria (Medical University of Vienna, *n* = 119; Medical University of Graz, *n* = 13; Medical University of Innsbruck: *n* = 256). Patient characteristics are depicted in Supplementary Table S4. sAxl and Gas6 (data not shown), as well as sAxl/alb and Gas6/alb ratios were increasing with Child Pugh Score (CPS) (Fig. 3A), model of end-stage liver disease (MELD) (Fig. 3B) as well as HVPG (Fig. 3C). Patients with decompensated cirrhosis (CPS B/C), ESLD (MELD ≥ 15), as well as CSPH (HVPG ≥ 10 mm Hg) had significantly higher levels of sAxl, Gas6, and their albumin ratios (Supplementary Table S5). Gas6/alb showed the best discriminatory potential for CPS B/C vs. CPS A (AUC 0.878), and MELD ≥ 15 vs. MELD < 15 (AUC 0.811), and equal discriminatory power as Gas6 for CSPH vs. no CSPH (AUC 0.778) (Supplementary Table S2). Using its respective optimal cut-offs of 20.70, 25.71, and 12.46, as depicted in Supplementary Table S2, the OR of Gas6/alb to show the probability of CPS B/C, MELD ≥ 15, and CSPH was 16.534 (95%CI: 9.972;27.415), 10.258 (95%CI: 6.045;17.406), and 12.115 (95%CI: 2.306;63.665), respectively.

### Probability of transplant-free survival

Furthermore, long-term follow-up data were available in 256 of 388 patients with liver cirrhosis. Of these patients, 151 (59.0%) had either CPS B or C, and 59 (23.0%) patients had a MELD ≥ 15 at the time of study inclusion. The median follow-up time was 7.4 y (Q1; Q3: 3.1;10.6). The median transplant-free survival (until liver transplantation or death) was 5.9 y (95%CI: 4.2;7.6).

Both sAxl and Gas6 were significantly associated with transplant-free survival in a univariable analysis, with Hazard ratios (HR) of 1.009 (95%CI: 1.006;1.011), and 1.014 (95%CI: 1.010;1.018), respectively. Using albumin ratios, i.e., sAxl/alb and Gas6/alb, their HR increased to 1.035 (95%CI: 1.026;1.044), and 1.031 (95%CI: 1.022;1.040), respectively, whereas, MELD score showed a HR of 1.094 (95%CI: 1.059;1.131). Patients with sAxl/alb ≥22.96 mg/dL (Youden Index = 0.342, sensitivity 64.8%, specificity 96.4%), and Gas6/alb ≥20.16 mg/dL (Youden Index = 0.249, sensitivity 71.7%, specificity 53.2%), respectively, had a significantly lower transplant-free survival (*p* < 0.001; Supplementary Fig. S1).

### sAxl and Gas6 levels to detect HCC

The HCC cohort was analyzed in order to evaluate sAxl and Gas6 as diagnostic biomarkers of HCC with and without cirrhosis. Furthermore, we determined if sAxl and Gas6 are specific for HCC by comparison to patients with HCC-free liver disease with and without cirrhosis, and to healthy volunteers, as well as to cholangiocarcinoma (CCA), and to colorectal liver metastases (CRCLM) (n = 1111). Patient characteristics are described in Supplementary Table S1. Briefly, 323 (29.1%) patients suffered from HCC, of these 267 (82.7%) developed HCC within a cirrhotic liver, whereas 56 (17.3%) showed HCCs in non-cirrhotic livers (Fig. 1). As controls, 36 (3.2%) patients with CCA, 54 (4.9%) patients with CRCLM, 698 patients with liver disease without HCC, and 57 healthy volunteers were included.

**Fig. 4:**
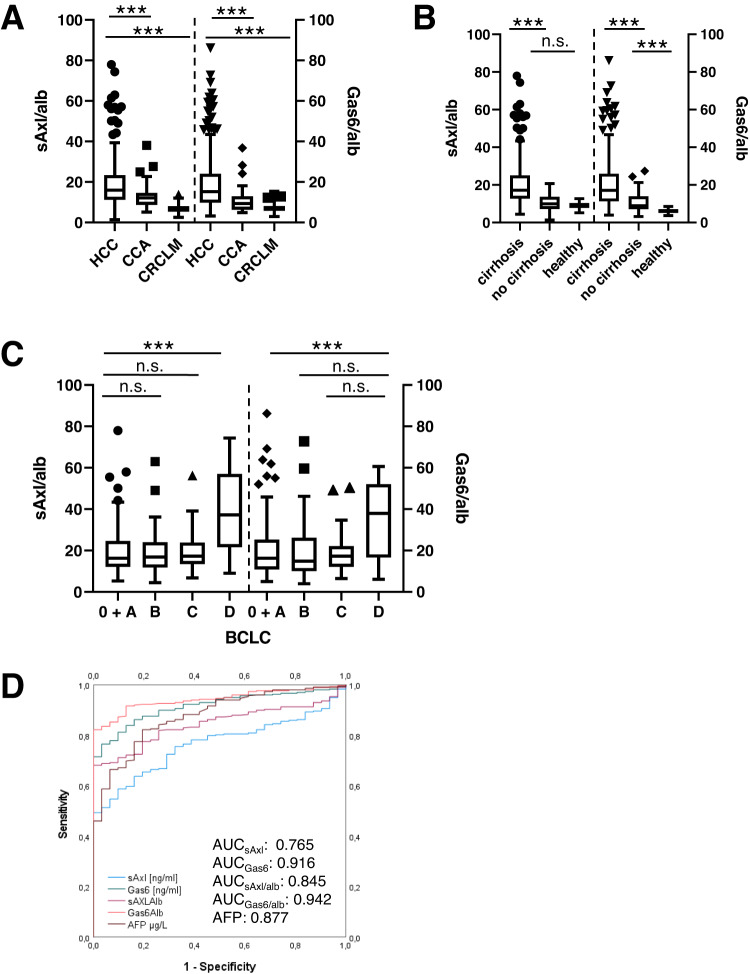
sAxl/alb and Gas6/alb in HCC patients. **A** sAxl/alb and Gas6/alb serum levels in HCC patients (*n* = 323) compared to CCA (*n* = 36) and CRCLM patients (*n* = 54). **B** Analysis of sAxl/alb and Gas6/alb serum levels in HCC patients with cirrhosis (*n* = 267) and HCC patients without cirrhosis (*n* = 56) and healthy controls (*n* = 57). **C** Analysis of sAxl/alb and Gas6/alb serum levels in HCC patients according to early BCLC stage (BCLC 0 + BCLC A, *n* = 128) and later BCLC stages (BCLC B, *n* = 81; BCLC C, *n* = 47, BCLC D, *n* = 15). **D** Comparison of the diagnostic accuracy shown as area under the curve (AUC) of sAxl/alb and Gas6/alb and sAxl and Gas6 in HCC versus healthy controls. Outliers are marked. HCC hepatocellular carcinoma, CCA cholangiocarcinoma, CRCLM colorectal carcinoma liver metastases, BCLC staging Barcelona Clinic Liver Cancer staging, n.s. not significant. Statistical significant differences are expressed as asterisks: ***P < 0.001.

sAxl and Gas6, as well as sAxl/alb and Gas6/alb were significantly higher in patients with HCC compared to patients with CCA or CRCLM (Supplementary Fig. S2A, Fig. 4A). sAxl and sAxl/alb, as well as Gas6 and Gas6/alb were significantly higher in patients with HCC and underlying cirrhosis as compared to HCC without cirrhosis (Fig. 4B, Supplementary Fig. S2B). Yet, Gas6 and Gas6/alb rather than sAxl and sAxl/alb were higher in HCC without cirrhosis versus healthy controls (Fig. 4B, Supplementary Fig. S2B). Moreover, both sAxl/alb and Gas6/alb ratios significantly increased during HCC progression by comparison of early BCLC stages (BCLC 0 + A) with late BCLC D (Fig. 4C), whereas changes in sAxl and Gas6 levels were non-significant (Supplementary Fig. S2C).

When comparing HCC patients in the presence or absence of liver cirrhosis to healthy controls, Gas6/alb achieved an excellent discriminatory power (AUC 0.942, 95% CI 0.917–0.968; Fig. 4D; Supplementary Table S2) superior to Gas6 (AUC 0.916, 95% CI 0.882–0.95), sAxl/alb (AUC 0.845, 95% CI 0.8–0.889), and sAxl (AUC 0.765, 95% CI 0.706–0.823; Fig. 4D, Supplementary Table S2). When HCC with cirrhosis was compared to patients with CLD without cirrhosis, good accuracy could be achieved for all markers similar to α-fetoprotein (AFP) (Supplementary Table S2). However, when comparing HCC with underlying liver cirrhosis versus cirrhosis without HCC, sAxl, Gas6 or their albumin ratios exhibited no discriminatory power (Supplementary Table S2).

## Discussion

In this study, we investigated the Axl ligand Gas6 and Gas6/alb for its value to accurately detect significant to advanced fibrosis, cirrhosis, and HCC, as well as its potential to predict decompensated cirrhosis, ESLD, CSPH, and transplant-free survival in a large multicenter cohort.

Gas/alb outperformed Gas6, sAxl, and sAxl/alb for the detection of biopsy-proven significant to advanced liver fibrosis and cirrhosis. For the detection of cirrhosis, Gas6/alb was even superior to FIB-4 and showed similar accuracy as ELF™ test. FIB-4 has recently been proposed as non-invasive screening test for CLD within the out-patient setting [8]. Notably, as FIB-4 includes only indirect markers of liver damage (AST, ALT), risk factors (age) or portal hypertension (platelets), it is no direct marker of liver fibrosis and should not be used as only decision tool [8, 19]. In contrast, Gas6/alb combines a direct marker of fibrosis (Gas6) and of liver function (albumin). Direct markers of fibrosis are associated with processes of extracellular matrix remodeling or measure serum markers that are upregulated during fibrogenesis, and have been incorporated in scores such as ELF™ test [8]. ELF™ test, although one of the most accurate non-invasive assessments for liver fibrosis [20], requires a special device for measurement and is linked to high costs, which limit its use to specialized liver centers [21, 22]. In contrast, Gas6 6 can be detected with a simple enzyme-linked immunosorbent assay (ELISA) at low cost and is combined with the routine parameter serum albumin.

Furthermore, we found that Gas/alb was an accurate biomarker for liver disease severity outperforming sAxl, sAxl/alb, and Gas6, as it was not only increasing with fibrosis stage, but also with CPS, MELD, and HVPG, and was able to non-invasively detect CSPH. Ultimately, sAxl, Gas6 and their albumin ratios were predictors of transplant-free survival. Further prospective studies are necessary to investigate the potential of Gas6/alb to predict liver-related outcome.

In addition, we confirmed that sAxl, Gas6, and their albumin ratios are specific for HCC in comparison to CCA and CRCLM. Their albumin ratios increased with BCLC stage and were good to excellent markers for HCC when compared with patients with non-cirrhotic CLD and healthy controls. However, no diagnostic power was observed in the detection of HCC within a cirrhotic or non-cirrhotic liver when compared to cirrhosis in the absence of malignancy (Supplementary Table S2). Most likely this can be justified by another feature of these biomarkers, namely their ability to detect liver fibrosis, which constitutes a major precondition for the development of HCC. Thus, patients with advanced fibrosis or cirrhosis exhibit already high levels of sAxl and Gas6 [23].

In this study, we used a dual Gas6 sandwich ELISA based on the AVB-S6-80 Axl decoy receptor as capture which binds to Gas6 with 80-fold higher affinity than the wild-type Axl receptor [24]. ELISA approaches focusing on the binding of AVB-S6-80 to either Gas6 or sAxl-Gas6 complexes revealed reliable detection of recombinant Gas6 rather than sAxl-Gas6 complexes that were generated in vitro (Binder, M., unpublished data). Thus, this modified Gas6 ELISA exclusively detects “free” Gas6 antigen that is not complexed with sAxl in blood serum. As Gas6 levels were found to be highly elevated in advanced fibrosis and cirrhosis (Fig. 2A) and progression of cirrhosis (Fig. 3), we conclude that excess of bioactive Gas6 is available to bind cognate Axl receptors. In the context of recent considerations on how Gas6/Axl acts in HSC activation as well as in HCC cells after Axl shedding [25], our data indicate that free Gas6 is abundantly available to stimulate Axl signaling.

Both Gas6 and Axl are expressed in Kupffer cells and liver sinusoidal endothelial cells under physiological conditions. Interestingly, Gas6/Axl signaling components are upregulated in the injured liver during HSC activation to myofibroblasts to prevent apoptosis [9], during malignant progression of neoplastic hepatocytes [26], and are drivers of tumor angiogenesis [27]. HIF-1α, AP1, and YAP/TAZ were reported to be involved in the transcriptional activation of Gas6/Axl [28]. As Axl is expressed in cancer cells of about 40% of HCC patients and suggested to have a crucial function in cancer invasion and epithelial to mesenchymal transition of HCC cells, the role of Axl shedding in HCC cells and activated HSCs/myofibroblasts remains an open issue. Yet, it is conceivable that post-translational control of Axl expression by receptor shedding—possibly triggered by ADAM10 and ADAM17 in liver cells—is less efficient and might not result in dampening of Axl signaling as synthesis of Axl exceeds. Shedded sAxl binding to Gas6 is inferior in capturing and escaping from Axl signaling. In addition, free Gas6 can even further stimulate the production of uncleaved Axl receptors in an autocrine feedback loop which facilitates activation of Axl signaling [25]. In this scenario, the high abundance of Gas6 and the larger ratio of free Gas6 to sAxl or free Gas6 to sAxl/Gas6 complexes explains why Gas6 represents the superior diagnostic biomarker when compared to sAxl.

The interpretation of our results is limited by the fact that Gas6/alb was though independent of BMI, but was significantly lower in male patients. Male sex was an independent predictor of Gas6/alb levels in patients with ≥F3, but not in patients with ≥F2. Future research on Gas6 and Gas6/alb levels in patients need to take sex differences into account. Although our cohort is large, it comprises a low number of patients with ALD. This may be due to the fact the patients with ALD often seek hepatologic care only late in their disease stage, or alcohol consumption remains underdiagnosed in a substantial proportion of NAFLD patients [29]. Yet, patients enrolled in this study represent a real-life cohort as patients were consecutively recruited from specialized centers.

In conclusion, Gas6/alb shows high accuracy for the detection of significant (≥F2) to advanced fibrosis (≥F3), and can predict the probability of decompensated cirrhosis, ESLD as measured by MELD, and CSPH. It shows excellent accuracy for the detection of biopsy-proven liver cirrhosis outperforming FIB-4. Further prospective studies are required to confirm the potential of Gas6/alb as screening marker for liver fibrosis and cirrhosis, and for the prediction of clinical outcome in patients with advanced chronic liver disease.

## Materials and methods

### Study population

Serum samples of consecutive male and female patients with CLD in the presence and absence of fibrosis, compensated and decompensated liver cirrhosis, HCC, cholangiocarcinoma (CCA), colorectal liver metastases (CRCLM), as well as healthy volunteers from five university medical centers in Germany and Austria were collected. Three study cohorts, i.e., a fibrosis cohort, a cirrhosis cohort, and an HCC cohort were built (Fig. 1).

The samples of the fibrosis cohort including healthy volunteers were collected as part of an Austrian project in Vienna and Graz aiming at biomarker development for NAFLD in comparison to CLDs of other origin (BioPersMed). Grading of liver fibrosis was performed according to liver histology based on Kleiner [30], Ludwig [31], or METAVIR classification [3] as appropriate. Only patients whose liver biopsy specimens showed ≥6 portal fields were included into the analysis. Liver biopsies were evaluated by two independent pathologists. Healthy subjects were characterized as described earlier [10]. sAxl as well as sAxl/alb ratio have already been investigated in the majority of this patient group, and the results have been recently published [10]. For investigating the value of Gas6 and Gas6/alb in this study, exclusively patients with biopsy-proven liver fibrosis or cirrhosis were included into the analysis.

Within the cirrhosis cohort, sAxl, Gas6, and their albumin ratios were investigated for their predictive potential concerning liver decompensation as well as transplant-free survival. The diagnosis of liver cirrhosis was performed according to liver imaging, clinical and laboratory parameters, and histology. Severity of liver cirrhosis was assessed according to Child Pugh Score (CPS [32], and Model of End-stage Liver Disease (MELD) score [33]. The results of invasive HVPG measurements were additionally available in a subgroup of patients.

Within the HCC cohort, biomarker test results of patients with or without liver cirrhosis were compared to patients with CCA, CRCLM, and the fibrosis cohort (CLD and healthy controls). The diagnosis of HCC, CCA, and CRCLM was performed by contrast-enhanced computed tomography or contrast-enhanced magnetic resonance imaging, and was confirmed by histology either after fine needle aspiration or resection.

Blood sampling including liver function tests was performed either within one day prior to surgery, liver biopsy, or respective imaging studies. Immediately after blood withdrawal, one sample of whole blood was centrifuged according to a standardized protocol and stored as serum aliquots at -80 ^◦^C for later analysis of sAxl, Gas6, and ELF™ levels.

### Assessment of sAxl and Gas6 in human sera

Serum levels of sAxl were detected by enzyme-linked immunosorbent assay (ELISA) as described recently [10, 18]. In order to analyze Gas6 levels in patient sera, the human Gas6 DuoSet® ELISA (R&D Systems, Minneapolis, USA) was used under optimized assay conditions essentially as described for detecting sAxl [34]. Changes in the ELISA assay conditions included the replacement of Gas6 capture antibody with the engineered Axl decoy receptor AVB-S6-80 (Aravive Biologics, Houston, USA) [24]. 500 ng/ml AVB-S6-80 were coated overnight on the microtiter plate. Serum samples were analyzed at a dilution of 1:200 in LowCross-buffer (Candor Bioscience, Wangen, Germany).

### Statistical analysis

Quantitative variables are expressed as median with first and third quartile (Q1;Q3) and were compared by Mann-Whitney U-tests. Qualitative variables are described using absolute and relative frequencies and were compared by Chi-Square- or Fisher´s exact tests, as appropriate. Comparison of more than two groups was done using the Kruskal–Wallis test. The diagnostic accuracy of sAxl and Gas6 as single markers and in combination with serum albumin levels (expressed as sAxl/alb ratio or Gas6/alb ratio) was assessed by areas under the curve (AUC) to calculate their discriminatory potential for (i) significant fibrosis (≥F2), advanced fibrosis (≥F3), and cirrhosis (F4) in comparison to FIB-4 and ELF™ test, (ii) decompensated cirrhosis defined by CPS B/C vs CPS A, end-stage liver disease (ESLD) defined by MELD ≥ 15 vs. MELD < 15, and CSPH defined by HVPG ≥ 10 mm Hg vs. <10 mm Hg, and (iii) detection of HCC in comparison to AFP. In order to identify optimal cut-off values were applicable, the Youden index was calculated [35]. In addition, sensitivity, specificity, positive predictive value (PPV), and negative predictive value (NPV) were computed.

Furthermore, a random sub-sampling cross-validation of diagnostic markers was performed. A training set containing 66% of the available data was randomly drawn to compute the optimal threshold (Youden index). The performance metrics were subsequently computed on the test set (containing the remaining 34% of the data). The random sub-sampling process was repeated 1000 times, resulting in an uncertainty distribution in the performance metrics which are summarized with mean and central 95% probability ranges.

Odds ratios (OR) were calculated to analyze the probability of CPS B/C, MELD ≥ 15, and CSPH in ESLD patients with increased sAxl, Gas6, and their albumin ratios. In addition, the predictive ability of sAxl, Gas6, and their albumin ratios for transplant-free survival was assessed by cox regression analysis.

*P*-values < 0.05 were considered statistically significant. Statistical analysis was performed using IBM Statistics SPSS 25.0 (IBM Corp., Armonk, NY) and R version 4.0.2 [36].

## Supplementary information

Supplementary Figure S1

Supplementary Figure S2

Supplementary Table S1

Supplementary Table S2

Supplementary Table S3

Supplementary Table S4

Supplementary Table S5

Supplementary Material legends

## Data Availability

All datasets generated and analyzed during this study are included in this published article and its Supplementary Information files. Additional data are available from the corresponding author on reasonable request.
