# Supplementary figures and images for: Gas6 in chronic liver disease—a novel blood-based biomarker for liver fibrosis
Source: Cell Death Discov. 2023 Aug 2;9:282. doi: 10.1038/s41420-023-01551-6 (PMC10397215; doi:10.1038/s41420-023-01551-6)

# Supplementary Figure 1

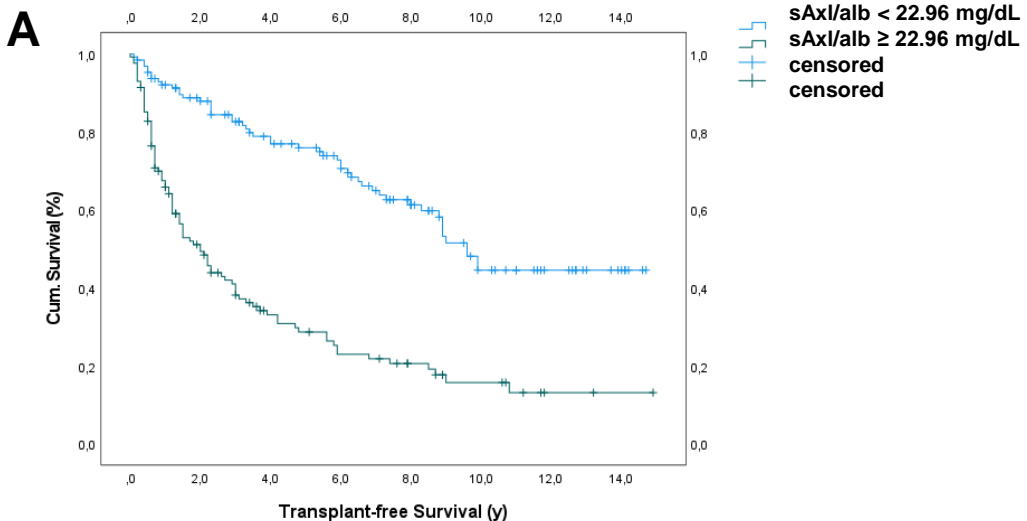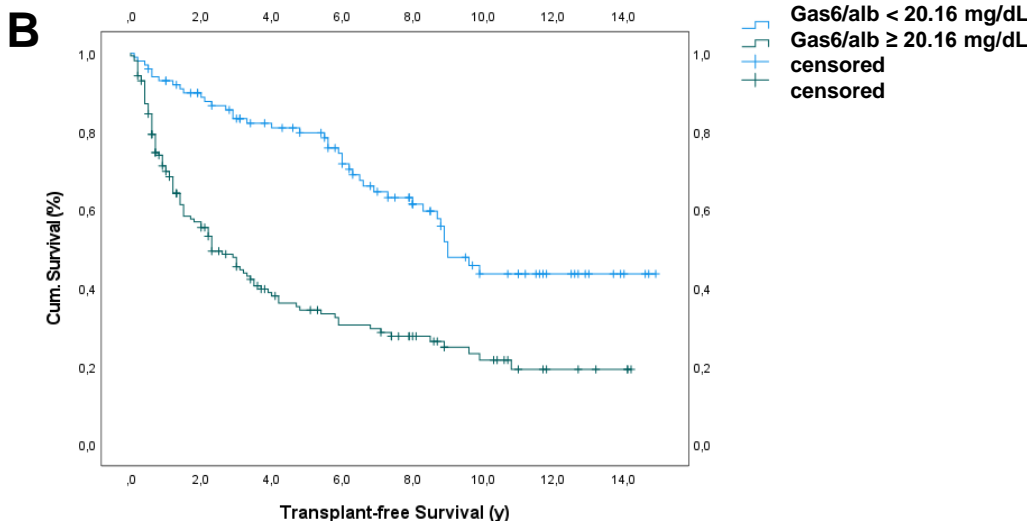

Supplement: Supplementary file 1 — Supplementary Figure S1 [file 41420_2023_1551_MOESM1_ESM.pdf]

Supplementary Figure 2

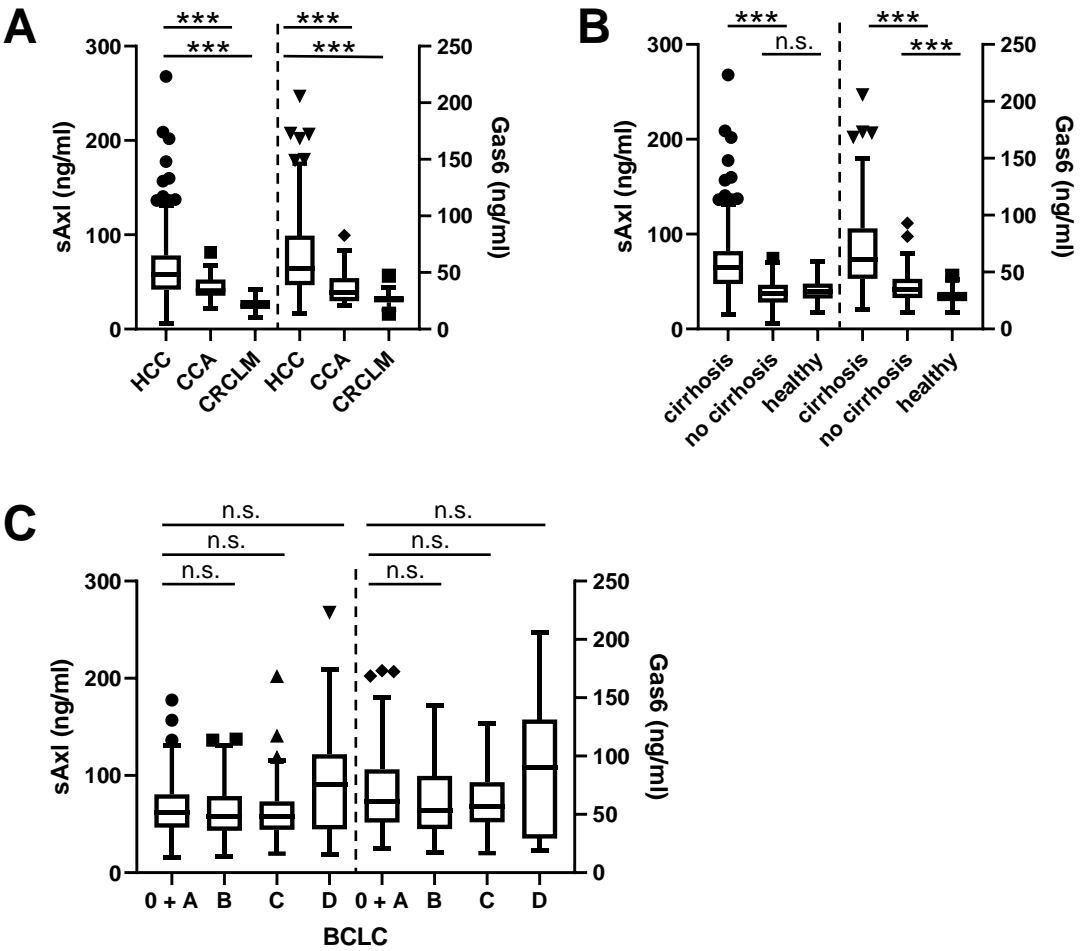

Supplement: Supplementary file 2 — Supplementary Figure S2 [file 41420_2023_1551_MOESM2_ESM.pdf]
