# Supplementary material for: Gas6 in chronic liver disease—a novel blood-based biomarker for liver fibrosis
Source: Cell Death Discov. 2023 Aug 2;9:282. doi: 10.1038/s41420-023-01551-6 (PMC10397215; doi:10.1038/s41420-023-01551-6)
Supplement: Supplementary file 3 — Supplementary Table S1 [file 41420_2023_1551_MOESM3_ESM.docx]

|  | **All patients**  **n=1111** | **Malignancy** | | | | **No malignancy** | | | |
| --- | --- | --- | --- | --- | --- | --- | --- | --- | --- |
|  |  | **All patients**  **n=413** | **HCC**  **n=323** | **CCA**  **n=36** | **CRCLM**  **n=54** | **All patients**  **n=698** | **Liver disease with cirrhosis**  **n=388** | **Liver disease without cirrhosis**  **n=253** | **Healthy controls**  **N=57** |
| Age (y); median (Q1;Q3) | 57.8 (48.4;66.5) | 64.1 (57.0;71.6) | 64.5 (58.0;71.1) | 63.8 (56.1;72.8) | 62.2 (52.9;71.8) | 53.7 (42.9;62.0) | 57.2 (49.1;64.7) | 48.3 (35.8;57.6) | 41.2 (30.4;53.5) |
| Male sex; n (%) | 748 (67.3) | 330 (79.9) | 277 (85.8) | 21 (58.3) | 32 (59.3) | 418 (59.9) | 260 (67.0) | 129 (51.0) | 29 (50.9) |
| BMI (kg/m^2^) median (Q1;Q3), n=989  Missing n=122 | 26.5 (23.6;30.1) | 26.7 (24.1;29.8),  n=366 | 27.1 (24.2;30.0) | 26.2 (24.8;31.9),  n=32 | 26.4 (23.0;28.7) | 26.3 (23.3;30.5) | 26.1 (23.0;29.3),  n=339 | 27.3 (24.2;33.3) | 23.3 (20.6;26.1) |
| *Liver disease etiology, n (%)*  NAFLD  Viral hepatitis  AIH/PSC/PBC/Overlap  DILI  Cryptogenic  Genetic disease  Other  unknown | 1000 (90)  437 (43.7)  270 (27.0)  67 (6.7)  8 (0.8)  54 (5.4)  7 (0.7)  14 (1.4)  143 (14.3) | 324 (78.5)  98 (23.7)  95 (23.0)  0 (0)  0 (0)  20 (4.8)  3 (0.7)  0 (0)  108 (26.2) | 323 (100)  98 (30.3)  94 (29.1)  0 (0)  0 (0)  20 (6.2)  3 (0.9)  0 (0)  108 (33.4) | 1 (2.8)  0 (0)  1 (2.8)  0 (0)  0 (0)  0 (0)  0 (0)  0 (0)  0 (0) | 0 (0) | 641 (91.8)  339 (48.7)  175 (25.1)  67 (9.6)  8 (1.1)  34 (4.9)  4 (0.6)  14 (2.0)  0 (0) | 388 (100)  206 (53.1)  109 (28.1)  33 (8.5)  0 (0)  27 (7.0)  4 (1.0)  9 (2.3)  0 (0) | 253 (100)  133 (52.6)  66 (26.1)  34 (13.4)  8 (3.2)  7 (2.8)  0 (0)  5 (2.0)  0 (0) | 0 (0) |
| Liver cirrhosis  CPS (stage), n=531  A  B  C  Missing  MELD, n=614 | 656 (57.3)  159 (24.2)  176 (26.8)  279 (42.4)  125 (19.1)  11.0 (8.0;14.0) | 267 (64.6)  62 (15.0)  76 (18.4)  88 (21.3)  97 (23.5)  10.0 (8.0;13.0) | 267 (82.7)  62 (19.2)  76 (23.5)  88 (27.2)  97 (30.0)  10.0 (8.0;13.0) | 1 (2.8)  n.a.  n.a. | 0 (0)  n.a.  n.a. | 388 (55.6)  97 (13.9)  100 (14.3)  191 (27.4)  310 (44.4)  11.0 (8.0;15.0) | 388 (100)  97 (25.0)  100 (25.8)  191 (49.2)  0 (0)  11.0 (8.0;15.0) | 0 (0)  n.a. | 0 (0)  n.a. |
| HCC, n (%)  Cirrhosis  No cirrhosis | 323 (28.2)  267 (82.7)  56 (17.3) | 323 (78.2)  267 (64.6)  56 (13.6) | 267 (82.7)  56 (17.3) | n.a. | 0 (0)  0 (0)  0 (0) | 0 (0)  388 (55.6)  310 (44.4) | 0 (0)  388 (100)  0 (0) | n.a. | 0 (0) |
| HCC – BCLC, n=323  0  A  B  C  D  missing |  |  | 6 (1.9)  122 (37.8)  81 (25.1)  47 (14.6)  15 (4.6)  52 (16.1) | n.a. | n.a. | n.a. | n.a. | n.a. | n.a. |
| Malignancy other than HCC  CCA  CRCLM | 90 (7.8)  36 (3.1)  54 (4.7) | 90 (21.8)  36 (8.7)  54 (13.1) | 0 (0) | 0 (0) | 0 (0) | 0 (0)  0 (0)  0 (0) | 0 (0)  0 (0)  0 (0) | 0 (0)  0 (0)  0 (0) | 0 (0) |
| AFP (kU/L), n=834 | 4.6 (2.7;10.7) | 9.4 (4.0;59.6),  n=341 | 10.7 (4.4;90.2) | n.a. | n.a. | 3.6 (2.2;6.0)  n=493 | 4.5 (2.7;7.1) | 2.5 (1.8;3.9), n=158 | n.a. |
| sAxl (ng/mL) | 55.00 (39.42;78.69) | 50.05 (34.48;72.42), | 57.89 (41.91;78.16) | 41.24 (35.29;52.72) | 25.03 (21.44;30.85) | 56.89 (41.20;82.15) | 76.40 (56.30;102.77) | 43.4  (35.6;55.8) | 39.92  (32.0;48.0) |
| sAXL/Alb, n=1053 | 14.08 (9.29;22.86) | 13.97 (8.79;21.52),  n=391 | 15.93 (11.23;23.35), n=309 | 12.11 (8.67;14.61) | 6.67 (5.35;8.04) | 14.23 (9.61;24.23) | 21.65 (14.52;30.08), n=380 | 9.82 (7.93;12.80), n=251 | 8.55 (7.75;10.40), n=31 |
| Gas 6 | 47.01 (31.69;80.59) | 46.30 (31.68;75.81) | 53.70 (38.76;82.67) | 32.60 (24.60;45.18) | 26.33  (23.75;29.32) | 47.88 (31.63;84.26) | 77.25 (52.78;104.83) | 32.59 (25.87;41.75) | 28.46 (24.47;32.69) |
| Gas6/Alb, n=1053 | 12.62 (7.54;22.86) | 12.88 (7.97;22.28),  n=391 | 15.17  (9.76;24.02), n=309 | 9.39 (6.28;12.93) | 6.54  (5.77;8.16) | 12.13 (7.26;23.33) | 21.34 (13.06;31.98), n=380 | 7.39  (5.88;9.59), n=251 | 6.37  (5.16;7.00), n=31 |
| ELF, n= 338 | 9.25 (8.39;10.69) | n.a. | n.a. | n.a. | n.a. | 9.27 (8.39;10.69), n=335 | 11.45 (10.3;12.40) | 8.82 (8.18;9.62), n=212 | 8.65 (7.99;9.27), n=27 |
| HVPG (mmHg), n=81 | 13.0 (8.0;19.0) | n.a. | n.a. | n.a. | n.a. | 13.5 (8.0;19.0), n=76 | 15.0 (10.0;20.0), n=66 | 4.5 (2.0;6.3), n=10 | n.a. |

**Supplementary Table S1. Patient characteristics of all patients including HCC cohort.** Patients with HCC were compared to patients with CCA, CRCLM, liver disease with and without cirrhosis, as well as healthy volunteers. HCC, hepatocellular carcinoma; CCA, cholangiocarcinoma; CRCLM, colorectal liver metastases; BMI, body mass index; NAFLD, non-alcoholic fatty liver disease; AIH, autoimmune hepatitis; PSC, primary sclerosing cholangitis; PBC, primary biliary cholangitis; DILI, drug induced liver injury; CPS, Child Pugh Score; MELD, Model of End-stage Liver Disease; BCLC, Barcelona Clinic Liver Cancer.
