# Supplementary material for: Gas6 in chronic liver disease—a novel blood-based biomarker for liver fibrosis
Source: Cell Death Discov. 2023 Aug 2;9:282. doi: 10.1038/s41420-023-01551-6 (PMC10397215; doi:10.1038/s41420-023-01551-6)
Supplement: Supplementary file 4 — Supplementary Table S2 [file 41420_2023_1551_MOESM4_ESM.docx]

| Fibrosis cohort, n = 333 |  | AUC  (95% CI) | Sensitivity (%) | Specificity (%) | PPV (%) | NPV (%) | Youden  Index | Cut-off (mg/dl) |
| --- | --- | --- | --- | --- | --- | --- | --- | --- |
| sAxl | ≥F2 | 0.734(0.678-0.789) | 59.2 | 78.7 | 74.4 | 64.8 | 0.379 | 52.78 |
|  | ≥F3 | 0.74(0.678-0.803) | 70.7 | 72.1 | 52.0 | 85.2 | 0.427 | 52.78 |
|  | F4 | 0.795(0.728-0.862) | 78.7 | 70.3 | 39.7 | 93.0 | 0.490 | 52.98 |
| sAxl/alb | ≥F2 | 0.756(0.703-0.81) | 54.1 | 86.7 | 81.0 | 64.4 | 0.408 | 12.89 |
|  | ≥F3 | 0.766(0.705-0.826) | 72.8 | 73.5 | 54.0 | 86.3 | 0.463 | 11.80 |
|  | F4 | 0.821(0.756-0.885) | 78.7 | 77.2 | 46.2 | 93.6 | 0.559 | 12.92 |
| Gas6 | ≥F2 | 0.79(0.74-0.841) | 67.5 | 78.0 | 76.3 | 69.6 | 0.455 | 37.14 |
|  | ≥F3 | 0.806(0.753-0.859) | 76.1 | 72.1 | 53.8 | 87.6 | 0.482 | 38.07 |
|  | F4 | 0.888(0.844-0.932) | 77.0 | 86.6 | 58.8 | 93.8 | 0.636 | 49.99 |
| Gas6/alb | ≥F2 | 0.805(0.756-0.854) | 74.5 | 76.0 | 76.5 | 74.0 | 0.505 | 7.94 |
|  | ≥F3 | 0.818(0.766-0.869) | 82.6 | 68.4 | 52.8 | 90.2 | 0.510 | 8.30 |
|  | F4 | 0.897(0.856-0.938) | 88.5 | 76.0 | 47.8 | 96.4 | 0.645 | 9.61 |
| ELF™ | ≥F2 | 0.822(0.771-0.874) | 76.0 | 77.0 | 77.2 | 75.8 | 0.530 | 9.15 |
|  | ≥F3 | 0.86(0.811-0.908) | 73.0 | 84.5 | 65.9 | 88.4 | 0.575 | 9.88 |
|  | F4 | 0.904(0.863-0.945) | 89.1 | 78.5 | 47.7 | 97.0 | 0.676 | 9.82 |
| FIB-4 | ≥F2 | 0.823(0.776-0.869) | 70.1 | 80.7 | 79.1 | 72.0 | 0.507 | 1.44 |
|  | ≥F3 | 0.838(0.79-0.886) | 82.6 | 72.1 | 55.9 | 90.6 | 0.547 | 1.45 |
|  | F4 | 0.878(0.832-0.925) | 82.0 | 79.7 | 50.0 | 94.7 | 0.616 | 1.98 |
| Cirrhosis cohort, n=388 |  | AUC  (95% CI) | Sensitivity (%) | Specificity (%) | PPV (%) | NPV (%) | Youden  Index | Cut-off (mg/dl) |
| sAxl | CPS B/C | 0.771(0.724-0.817) | 77.9 | 64.9 | 70.0 | 73.6 | 0.428 | 72.34 |
|  | MELD ≥ 15 | 0.768(0.715-0.82) | 61.5 | 79.7 | 53.3 | 84.6 | 0.412 | 92.14 |
|  | HVPG ≥ 10 mmHg, n=59 | 0.667(0.47-0.863) | 64.6 | 81.8 | 93.9 | 34.6 | 0.464 | 55.41 |
| sAxl/alb | CPS B/C | 0.85(0.812-0.888) | 84.1 | 73.5 | 77.0 | 81.4 | 0.576 | 19.60 |
|  | MELD ≥ 15 | 0.803(0.754-0.852) | 85.6 | 64.9 | 47.8 | 92.3 | 0.504 | 21.82 |
|  | HVPG ≥ 10 mmHg, n=59 | 0.701(0.514-0.887) | 47.9 | 90.9 | 95.8 | 28.6 | 0.388 | 19.06 |
| Gas6 | CPS B/C | 0.838(0.798-0.878) | 71.3 | 83.2 | 81.8 | 73.3 | 0.545 | 83.38 |
|  | MELD ≥ 15 | 0.808(0.758-0.859) | 77.9 | 71.0 | 50.3 | 89.5 | 0.489 | 86.88 |
|  | HVPG ≥ 10 mmHg, n=59 | 0.778(0.625-0.932) | 68.8 | 81.8 | 94.3 | 37.5 | 0.506 | 47.26 |
| Gas6/alb | CPS B/C | 0.878(0.844-0.912) | 81.5 | 78.9 | 80.3 | 80.2 | 0.605 | 20.70 |
|  | MELD ≥ 15 | 0.811(0.76-0.861) | 76.0 | 76.4 | 54.9 | 89.4 | 0.524 | 25.71 |
|  | HVPG ≥ 10 mmHg, n=59 | 0.778(0.623-0.934) | 72.9 | 81.8 | 94.6 | 40.9 | 0.547 | 12.46 |
| HCC, n= 323 |  | AUC  (95% CI) | Sensitivity (%) | Specificity (%) | PPV (%) | NPV (%) | Youden  Index | Cut-off (mg/dl) |
| sAxl | HCC w cirrhosis vs. cirrhosis w/o HCC | 0.355 (0.309;0.400 | 59.9 | 64.5 | 58.3 | 66.0 | 0.244 | 70.72 |
|  | HCC w cirrhosis vs. HCC w/o cirrhosis | 0.854(0.803-0.904) | 73.4 | 84.8 | 96.4 | 36.8 | 0.582 | 48.78 |
|  | HCC w cirrhosis vs. CLD w/o cirrhosis w/o HCC | 0.766(0.719-0.813) | 64.3 | 79.7 | 83.5 | 58.3 | 0.440 | 55.81 |
|  | HCC w/o cirrhosis vs. CLD w/o cirrhosis w/o HCC | 0.368 (0.275;0.461) | 50.0 | 75.3 | 37.1 | 83.8 | 0.253 | 34.20 |
|  | HCC (all) vs. healthy controls | 0.765(0.706-0.823) | 49.3 | 100.0 | 100.0 | 17.0 | 0.493 | 60.25 |
| sAxl/alb | HCC w cirrhosis vs. cirrhosis w/o HCC | 0.367 (0.321;0.413) | 56.7 | 65.1 | 57.4 | 64.5 | 0.219 | 18.49 |
|  | HCC w cirrhosis vs. HCC w/o cirrhosis | 0.81(0.749-0.872) | 81.3 | 69.6 | 93.6 | 40.5 | 0.509 | 11.54 |
|  | HCC w cirrhosis vs. CLD w/o cirrhosis w/o HCC | 0.814(0.771-0.857) | 79.8 | 76.6 | 84.5 | 70.3 | 0.563 | 11.88 |
|  | HCC w/o cirrhosis vs. CLD w/o cirrhosis w/o HCC | 0.504(0.403-0.606) | 39.1 | 69.6 | 27.3 | 79.7 | 0.088 | 11.30 |
|  | HCC (all) vs. healthy controls | 0.845(0.8-0.889) | 68.1 | 100.0 | 100.0 | 24.6 | 0.681 | 12.68 |
| Gas6 | HCC w cirrhosis vs. cirrhosis w/o HCC | 0.633(0.587-0.679) | 67.1 | 54.6 | 55.0 | 66.7 | 0.217 | 80.42 |
|  | HCC w cirrhosis vs. HCC w/o cirrhosis | 0.841(0.783-0.899) | 74.6 | 82.6 | 95.9 | 37.3 | 0.572 | 45.21 |
|  | HCC w cirrhosis vs. CLD w/o cirrhosis w/o HCC | 0.852(0.813-0.891) | 84.1 | 75.9 | 84.8 | 75.0 | 0.601 | 39.64 |
|  | HCC w/o cirrhosis vs. CLD w/o cirrhosis w/o HCC | 0.554(0.459-0.648) | 58.7 | 59.5 | 29.7 | 83.2 | 0.182 | 33.66 |
|  | HCC (all) vs. healthy controls | 0.916(0.882-0.95) | 76.5 | 96.8 | 99.6 | 30.0 | 0.733 | 38.56 |
| Gas6/alb | HCC w cirrhosis vs. cirrhosis w/o HCC | 0.391 (0.344;0.438) | 61.5 | 57.6 | 54.6 | 64.3 | 0.191 | 20.64 |
|  | HCC w cirrhosis vs. HCC w/o cirrhosis | 0.787(0.719-0.854) | 76.2 | 69.6 | 93.2 | 34.8 | 0.458 | 11.23 |
|  | HCC w cirrhosis vs. CLD w/o cirrhosis w/o HCC | 0.868(0.831-0.904) | 81.7 | 81.0 | 87.3 | 73.6 | 0.628 | 9.97 |
|  | HCC w/o cirrhosis vs. CLD w/o cirrhosis w/o HCC | 0.65(0.56-0.74) | 80.4 | 45.6 | 30.1 | 88.9 | 0.260 | 6.99 |
|  | HCC (all) vs. healthy controls | 0.942(0.917-0.968) | 82.2 | 100.0 | 100.0 | 36.9 | 0.822 | 8.66 |
| AFP | HCC w cirrhosis vs. cirrhosis w/o HCC | 0.759(0.719-0.8) | 68.3 | 74.7 | 69.1 | 73.9 | 0.429 | 6.85 |
|  | HCC w cirrhosis vs. HCC w/o cirrhosis | 0.639(0.547-0.73) | 73.4 | 54.3 | 89.8 | 27.2 | 0.278 | 5.50 |
|  | HCC w cirrhosis vs. CLD w/o cirrhosis w/o HCC | 0.869(0.835-0.904) | 77.4 | 83.5 | 88.2 | 69.8 | 0.609 | 4.87 |
|  | HCC w/o cirrhosis vs. CLD w/o cirrhosis w/o HCC | 0.773(0.696-0.85) | 87.0 | 53.2 | 35.1 | 93.3 | 0.401 | 2.79 |
|  | HCC (all) vs. healthy controls | 0.877(0.824-0.929) | 82.2 | 80.6 | 97.6 | 32.1 | 0.629 | 3.66 |
|  |  |  |  |  |  |  |  |  |

Supplemenary Table S2. Diagnostic accuracy of biomarkers for HCC, liver fibrosis grade, and end-stage liver disease. AUC, area under the curve; CI, confidence interval; PPV, positive predictive value; NPV, negative predictive value; YI, Youden Index; HCC, hepatocellular carcinoma; alb, albumin; AFP, alpha-fetoprotein; CI, confidence interval; w/o, without; F, fibrosis grade; ELF™, enhanced liver fibrosis test.
