# Supplementary material for: Gas6 in chronic liver disease—a novel blood-based biomarker for liver fibrosis
Source: Cell Death Discov. 2023 Aug 2;9:282. doi: 10.1038/s41420-023-01551-6 (PMC10397215; doi:10.1038/s41420-023-01551-6)
Supplement: Supplementary file 5 — Supplementary Table S3 [file 41420_2023_1551_MOESM5_ESM.docx]

| **Fibrosis cohort, n = 333** | **AUC** | **YI** | **Cutoff** | **SENS** | **SPEC** | **PPV** | **NPV** |
| --- | --- | --- | --- | --- | --- | --- | --- |
|  | | | | | | | |
| **sAxl [ng/ml]** | | | | | | | |
| >=F2 | 0.733 (0.694-0.776) | 0.387 (0.318-0.457) | 51.39 (42.84-56.20) | 0.745 (0.508-0.882) | 0.582 (0.435-0.760) | 0.630 (0.540-0.720) | 0.714 (0.573-0.842) |
| >=F3 | 0.740 (0.695-0.786) | 0.428 (0.352-0.507) | 53.00 (52.89-55.27) | 0.719 (0.623-0.808) | 0.682 (0.518-0.818) | 0.840 (0.762-0.915) | 0.512 (0.385-0.640) |
| F4 | 0.794 (0.746-0.842) | 0.507 (0.425-0.592) | 57.13 (52.98-65.82) | 0.761 (0.627-0.895) | 0.671 (0.417-0.889) | 0.906 (0.844-0.968) | 0.419 (0.263-0.600) |
| **sAxl/alb** | | | | | | | |
| >=F2 | 0.756 (0.717-0.795) | 0.419 (0.355-0.487) | 11.87 (9.76-13.75) | 0.767 (0.530-0.936) | 0.585 (0.407-0.789) | 0.643 (0.545-0.750) | 0.740 (0.592-0.895) |
| >=F3 | 0.767 (0.727-0.812) | 0.470 (0.395-0.546) | 12.05 (11.84-12.94) | 0.742 (0.657-0.838) | 0.687 (0.515-0.833) | 0.847 (0.768-0.922) | 0.534 (0.395-0.667) |
| F4 | 0.821 (0.776-0.867) | 0.567 (0.487-0.651) | 13.45 (12.94-16.17) | 0.790 (0.694-0.916) | 0.719 (0.444-0.900) | 0.920 (0.857-0.972) | 0.463 (0.324-0.639) |
| **Gas6 [ng/ml]** | | | | | | | |
| >=F2 | 0.791 (0.754-0.826) | 0.469 (0.401-0.536) | 37.71 (33.39-47.56) | 0.746 (0.562-0.918) | 0.651 (0.451-0.826) | 0.678 (0.567-0.795) | 0.736 (0.612-0.875) |
| >=F3 | 0.806 (0.768-0.842) | 0.487 (0.416-0.559) | 38.32 (33.39-50.08) | 0.693 (0.500-0.875) | 0.734 (0.455-0.926) | 0.865 (0.765-0.952) | 0.508 (0.379-0.652) |
| F4 | 0.887 (0.858-0.918) | 0.643 (0.571-0.720) | 47.59 (38.57-53.27) | 0.819 (0.651-0.920) | 0.765 (0.571-0.950) | 0.935 (0.886-0.983) | 0.526 (0.344-0.714) |
| **Gas6/alb** | | | | | | | |
| >=F2 | 0.806 (0.769-0.845) | 0.512 (0.440-0.578) | 8.21 (7.53-9.65) | 0.759 (0.588-0.889) | 0.705 (0.519-0.830) | 0.712 (0.603-0.821) | 0.758 (0.650-0.860) |
| >=F3 | 0.818 (0.781-0.858) | 0.519 (0.449-0.591) | 8.62 (7.95-9.89) | 0.696 (0.577-0.826) | 0.763 (0.543-0.917) | 0.876 (0.788-0.957) | 0.518 (0.400-0.643) |
| >=F4 | 0.897 (0.866-0.928) | 0.662 (0.586-0.736) | 10.26 (9.37-12.90) | 0.782 (0.679-0.892) | 0.808 (0.579-0.962) | 0.944 (0.882-0.987) | 0.483 (0.345-0.643) |
| **Cirrhosis cohort, n=388** | **AUC** | **YI** | **Cutoff** | **SENS** | **SPEC** | **PPV** | **NPV** |
| **sAxl [ng/ml]** | | | | | | | |
| CPS B/C | 0.774 (0.742-0.807) | 0.441 (0.379-0.503) | 72.86 (65.81-82.04) | 0.654 (0.531-0.778) | 0.752 (0.554-0.853) | 0.720 (0.612-0.818) | 0.693 (0.600-0.773) |
| MELD >= 15 | 0.767 (0.733-0.804) | 0.426 (0.362-0.496) | 88.76 (72.53-102.93) | 0.738 (0.483-0.865) | 0.620 (0.432-0.853) | 0.840 (0.775-0.909) | 0.483 (0.333-0.625) |
| HVPG >= 10 mmHg | 0.657 (0.533-0.791) | 0.474 (0.321-0.649) | 56.59 (56.45-58.03) | 0.827 (0.333-1.000) | 0.601 (0.412-0.789) | 0.332 (0.100-0.556) | 0.935 (0.800-1.000) |
| **sAxl/alb** | | | | | | | |
| CPS B/C | 0.850 (0.822-0.878) | 0.581 (0.520-0.639) | 19.94 (19.32-21.13) | 0.731 (0.631-0.821) | 0.820 (0.712-0.906) | 0.795 (0.687-0.887) | 0.764 (0.687-0.841) |
| MELD >= 15 | 0.803 (0.769-0.839) | 0.510 (0.447-0.570) | 22.32 (21.13-26.49) | 0.654 (0.553-0.789) | 0.808 (0.548-0.939) | 0.903 (0.812-0.969) | 0.468 (0.369-0.571) |
| HVPG >= 10 mmHg | 0.698 (0.564-0.839) | 0.449 (0.270-0.640) | 16.10 (10.78-20.37) | 0.618 (0.000-1.000) | 0.568 (0.267-0.933) | 0.250 (0.000-0.500) | 0.882 (0.714-1.000) |
| **Gas6 [ng/ml]** | | | | | | | |
| CPS B/C | 0.838 (0.810-0.864) | 0.549 (0.494-0.607) | 79.19 (66.46-86.62) | 0.779 (0.594-0.894) | 0.730 (0.596-0.881) | 0.741 (0.643-0.852) | 0.778 (0.656-0.879) |
| MELD >= 15 | 0.801 (0.765-0.839) | 0.495 (0.429-0.562) | 87.06 (76.74-102.84) | 0.708 (0.559-0.879) | 0.730 (0.486-0.900) | 0.879 (0.804-0.948) | 0.492 (0.373-0.656) |
| HVPG >= 10 mmHg | 0.722 (0.601-0.848) | 0.473 (0.293-0.660) | 47.52 (31.09-52.10) | 0.616 (0.000-1.000) | 0.677 (0.444-0.941) | 0.319 (0.000-0.625) | 0.883 (0.733-1.000) |
| **Gas6/alb** | | | | | | | |
| CPS B/C | 0.877 (0.854-0.901) | 0.610 (0.553-0.665) | 21.30 (18.68-26.29) | 0.788 (0.651-0.945) | 0.780 (0.567-0.893) | 0.777 (0.645-0.884) | 0.800 (0.703-0.923) |
| MELD >= 15 | 0.811 (0.775-0.845) | 0.529 (0.457-0.595) | 26.68 (25.83-30.30) | 0.771 (0.691-0.859) | 0.718 (0.553-0.861) | 0.880 (0.817-0.941) | 0.543 (0.419-0.651) |
| HVPG >= 10 mmHg | 0.777 (0.660-0.893) | 0.554 (0.376-0.758) | 12.80 (9.02-13.99) | 0.745 (0.000-1.000) | 0.707 (0.500-0.933) | 0.374 (0.000-0.714) | 0.921 (0.737-1.000) |
| **HCC cohort, n=323** | **AUC** | **YI** | **Cutoff** | **SENS** | **SPEC** | **PPV** | **NPV** |
| **sAxl [ng/ml]** | | | | | | | |
| HCCwCIRRHvsCIRRHwoHCC | 0.384 (0.351-0.413) | 0.005 (0.004-0.007) | 161.78 (17.23-267.80) | 0.588 (0.000-0.993) | 0.399 (0.000-0.990) | 0.354 (0.000-0.643) | 0.166 (0.000-0.445) |
| HCCwCIRRHvsHCCwoCIRRH | 0.835 (0.800-0.871) | 0.571 (0.498-0.647) | 48.36 (46.78-49.71) | 0.799 (0.600-0.944) | 0.728 (0.638-0.813) | 0.381 (0.263-0.500) | 0.946 (0.892-0.986) |
| HCCwCIRRHvsCLDwoCIRHHwoHCC | 0.734 (0.701-0.765) | 0.387 (0.335-0.439) | 56.93 (45.82-64.95) | 0.742 (0.511-0.894) | 0.588 (0.441-0.794) | 0.630 (0.551-0.730) | 0.721 (0.602-0.839) |
| HCCwoCIRHHvsCLDwoCIRRHwoHCC | 0.382 (0.314-0.445) | 0.012 (0.001-0.046) | 29.14 (21.32-58.75) | 0.130 (0.000-0.724) | 0.636 (0.000-0.881) | 0.290 (0.000-0.749) | 0.146 (0.000-0.231) |
| HCCvsHealthy | 0.764 (0.726-0.798) | 0.485 (0.431-0.550) | 53.96 (43.10-60.42) | 0.856 (0.538-1.000) | 0.551 (0.415-0.712) | 0.252 (0.167-0.338) | 0.959 (0.889-1.000) |
| **sAxl/alb** | | | | | | | |
| HCCwCIRRHvsCIRRHwoHCC | 0.403 (0.375-0.434) | 0.010 (0.002-0.024) | 49.39 (5.14-77.98) | 0.772 (0.000-0.993) | 0.207 (0.000-0.989) | 0.500 (0.000-0.643) | 0.247 (0.000-0.500) |
| HCCwCIRRHvsHCCwoCIRRH | 0.806 (0.762-0.850) | 0.509 (0.412-0.604) | 12.50 (11.55-13.97) | 0.695 (0.471-0.883) | 0.755 (0.621-0.851) | 0.368 (0.229-0.519) | 0.925 (0.873-0.973) |
| HCCwCIRRHvsCLDwoCIRHHwoHCC | 0.806 (0.780-0.833) | 0.523 (0.474-0.574) | 13.07 (11.76-13.56) | 0.767 (0.630-0.867) | 0.721 (0.616-0.830) | 0.726 (0.643-0.810) | 0.765 (0.670-0.851) |
| HCCwoCIRHHvsCLDwoCIRRHwoHCC | 0.486 (0.422-0.554) | 0.105 (0.030-0.189) | 14.78 (10.14-16.89) | 0.810 (0.471-0.951) | 0.203 (0.045-0.476) | 0.823 (0.746-0.890) | 0.213 (0.038-0.429) |
| HCCvsHealthy | 0.839 (0.807-0.871) | 0.678 (0.639-0.722) | 12.55 (10.79-12.78) | 0.941 (0.571-1.000) | 0.676 (0.598-0.774) | 0.224 (0.116-0.324) | 0.992 (0.941-1.000) |
| **Gas6 [ng/ml]** | | | | | | | |
| HCCwCIRRHvsCIRRHwoHCC | 0.408 (0.380-0.439) | 0.008 (0.000-0.024) | 44.28 (20.53-206.02) | 0.122 (0.000-0.977) | 0.830 (0.000-0.977) | 0.351 (0.000-0.589) | 0.361 (0.000-0.450) |
| HCCwCIRRHvsHCCwoCIRRH | 0.836 (0.800-0.876) | 0.574 (0.502-0.657) | 45.50 (40.13-52.16) | 0.777 (0.526-0.952) | 0.719 (0.563-0.849) | 0.372 (0.250-0.516) | 0.941 (0.884-0.986) |
| HCCwCIRRHvsCLDwoCIRHHwoHCC | 0.840 (0.816-0.863) | 0.560 (0.514-0.612) | 41.98 (39.73-48.51) | 0.744 (0.644-0.851) | 0.786 (0.632-0.874) | 0.766 (0.667-0.849) | 0.769 (0.696-0.842) |
| HCCwoCIRHHvsCLDwoCIRRHwoHCC | 0.531 (0.473-0.589) | 0.139 (0.049-0.239) | 33.78 (28.84-38.76) | 0.535 (0.304-0.723) | 0.526 (0.222-0.750) | 0.833 (0.750-0.912) | 0.202 (0.105-0.288) |
| HCCvsHealthy | 0.895 (0.871-0.919) | 0.714 (0.656-0.774) | 37.01 (34.02-39.73) | 0.879 (0.700-1.000) | 0.774 (0.685-0.867) | 0.410 (0.286-0.550) | 0.974 (0.937-1.000) |
| **Gas6/alb** | | | | | | | |
| HCCwCIRRHvsCIRRHwoHCC | 0.408 (0.380-0.439) | 0.008 (0.000-0.024) | 44.28 (20.53-206.02) | 0.122 (0.000-0.977) | 0.830 (0.000-0.977) | 0.351 (0.000-0.589) | 0.361 (0.000-0.450) |
| HCCwCIRRHvsHCCwoCIRRH | 0.836 (0.800-0.876) | 0.574 (0.502-0.657) | 45.50 (40.13-52.16) | 0.777 (0.526-0.952) | 0.719 (0.563-0.849) | 0.372 (0.250-0.516) | 0.941 (0.884-0.986) |
| HCCwCIRRHvsCLDwoCIRHHwoHCC | 0.840 (0.816-0.863) | 0.560 (0.514-0.612) | 41.98 (39.73-48.51) | 0.744 (0.644-0.851) | 0.786 (0.632-0.874) | 0.766 (0.667-0.849) | 0.769 (0.696-0.842) |
| HCCwoCIRHHvsCLDwoCIRRHwoHCC | 0.531 (0.473-0.589) | 0.139 (0.049-0.239) | 33.78 (28.84-38.76) | 0.535 (0.304-0.723) | 0.526 (0.222-0.750) | 0.833 (0.750-0.912) | 0.202 (0.105-0.288) |
| HCCvsHealthy | 0.895 (0.871-0.919) | 0.714 (0.656-0.774) | 37.01 (34.02-39.73) | 0.879 (0.700-1.000) | 0.774 (0.685-0.867) | 0.410 (0.286-0.550) | 0.974 (0.937-1.000) |
| **AFP [kU/L]** | | | | | | | |
| HCCwCIRRHvsCIRRHwoHCC | 0.761 (0.734-0.789) | 0.437 (0.389-0.488) | 7.66 (6.70-9.30) | 0.764 (0.677-0.860) | 0.643 (0.511-0.747) | 0.716 (0.638-0.787) | 0.701 (0.619-0.794) |
| HCCwCIRRHvsHCCwoCIRRH | 0.628 (0.561-0.694) | 0.282 (0.179-0.379) | 6.85 (4.00-11.80) | 0.506 (0.250-0.790) | 0.685 (0.443-0.832) | 0.230 (0.121-0.360) | 0.885 (0.823-0.945) |
| HCCwCIRRHvsCLDwoCIRHHwoHCC | 0.872 (0.848-0.895) | 0.618 (0.567-0.669) | 5.06 (3.90-5.80) | 0.823 (0.673-0.923) | 0.754 (0.655-0.857) | 0.672 (0.573-0.767) | 0.877 (0.798-0.945) |
| HCCwoCIRHHvsCLDwoCIRRHwoHCC | 0.776 (0.719-0.833) | 0.430 (0.343-0.524) | 3.95 (2.66-8.95) | 0.653 (0.423-0.963) | 0.656 (0.235-1.000) | 0.874 (0.766-1.000) | 0.387 (0.219-0.714) |
| HCCvsHealthy | 0.877 (0.838-0.915) | 0.640 (0.549-0.738) | 4.46 (3.30-6.40) | 0.789 (0.499-1.000) | 0.762 (0.602-0.877) | 0.260 (0.138-0.412) | 0.974 (0.937-1.000) |

**Supplementary Table S3. Diagnostic accuracy of biomarkers for HCC, liver fibrosis grade, and end-stage liver disease after balanced random sub-sampling cross-validation.** AUC, area under the curve; CI, confidence interval; PPV, positive predictive value; NPV, negative predictive value; YI, Youden Index; HCC, hepatocellular carcinoma; alb, albumin; AFP, alpha-fetoprotein; CI, confidence interval; w/o, without; F, fibrosis grade; ELF™, enhanced liver fibrosis test.
