# Supplementary material for: Gas6 in chronic liver disease—a novel blood-based biomarker for liver fibrosis
Source: Cell Death Discov. 2023 Aug 2;9:282. doi: 10.1038/s41420-023-01551-6 (PMC10397215; doi:10.1038/s41420-023-01551-6)
Supplement: Supplementary file 6 — Supplementary Table S4 [file 41420_2023_1551_MOESM6_ESM.docx]

| **Cirrhosis cohort** | **All patients**  **N=388** |
| --- | --- |
| Age (y); median (Q1;Q3) | 57.2 (49.1;64.7) |
| Male sex; n (%) | 260 (67) |
| BMI (kg/m^2^) median (Q1;Q3), n=339 | 26.1 (23.4;29.3) |
| Liver disease etiology, n (%)  NAFLD/ALD  Viral hepatitis  AIH/PSC/PBC/Overlap  Cryptogenic  Genetic disease | 206 (53.1)  109 (28.1)  33 (8.5)  27 (7.0)  4 (1.0) |
| CPS (stage)  A  B  C  CPS (score)  MELD  HVPG (mmHg), n=66  normal (3-5 mmHg) – increased (6-9 mmHg), n (%)  significantly increased (≥10 mmHg), n (%) | 191 (49.2)  172 (44.3)  25 (6.4)  7.0 (5.0;8.0)  11.0 (8.5;15.0)  15.0 (10.0-20.0)  13 (19.7)  53 (80.3) |
| Noninvasive biomarkers  sAxl (ng/ml)  sAxl/alb*10, n=380  Gas 6 (ng/ml)  Gas6/alb*10, n=380 | 76.4 (56.3;102.8)  21.7 (14.5;30.1)  77.3 (52.1;104.8)  21.3 (13.1;32.0) |

**Supplementary Table S4. Patient characteristics of patients with liver cirrhosis: Cirrhosis** **cohort.** None of the patients had any malignancy or benign liver tumor. BMI, body mass index; NAFLD, non-alcoholic steatohepatitis; ALD, alcoholic liver disease; AIH, autoimmune liver disease; PSC, primary sclerosing cholangitis; PBC, primary biliary cholangitis; CPS, Child Pugh Score; MELD, Model of End-Stage Liver Disease; HVPG, hepatic venous pressure gradient; alb, albumin.
