# Supplementary material for: Gas6 in chronic liver disease—a novel blood-based biomarker for liver fibrosis
Source: Cell Death Discov. 2023 Aug 2;9:282. doi: 10.1038/s41420-023-01551-6 (PMC10397215; doi:10.1038/s41420-023-01551-6)
Supplement: Supplementary file 7 — Supplementary Table S5 [file 41420_2023_1551_MOESM7_ESM.docx]

| **Cirrhosis cohort**  **n=388** | **CPS A**  **n=191** | **CPS B/C**  **n= 197** | **p-value** | **MELD < 15**  **n=280** | **MELD ≥ 15**  **n=108** | **p-value** | **HVPG <10 mmHg**  **n=13** | **HVPG ≥ 10 mmHg**  **n=53** | **p-value** |
| --- | --- | --- | --- | --- | --- | --- | --- | --- | --- |
| Age (y); median (Q1;Q3) | 56.7 (47.7;64.9) | 57.2 (50.7;64.6) | 0.451 | 57.6 (49.2;64.9) | 56.4 (48.9;64.2) | 0.423 | 54.9 (39.8;66.2) | 56.1 (43.49;59.3) | 0.442 |
| Male sex; n (%) | 124 (64.9) | 136 (69.0)  **A** | 0.389 | 186 (66.4) | 74 (68.5) | 0.695 | 7 (53.8) | 30 (56.6) | 0.858 |
| BMI (kg/m^2^) median (Q1;Q3), n=339 | 26.2 (23.4;29.7) | 25.9 (22.5;28.7) | 0.360 | 26.3 (23.5;30.0) | 24.8 (22.5;28.1) | 0.040 | 24.2 (23.3;30.1) | 25.2 (22.6;28.7) | 0.737 |
| Liver disease etiology, n (%)  NAFLD/ALD  Viral hepatitis  AIH/PSC/PBC/Overlap  Cryptogenic  Genetic disease  other | 84 (44.0)  70 (36.6)  19 (9.9)  11 (5.8)  1 (0.5)  6 (3.1) | 122 (61.9)  39 (19.8)  14 (7.1)  16 (8.1)  3 (1.5)  3 (1.5) | <0.001 | 139 (49.6)  91 (32.5)  26 (9.3)  16 (5.7)  1 (0.4)  7 (2.5) | 67 (62.0)  18 (16.7)  7 (6.5)  11 (10.2)  3 (2.8)  2 (1.9) | 0.008 | 4 (30.8)  3 (23.1)  3 (231)  0  0  3 (23.1) | 24 (45.3)  15 (28.3)  5 (9.4)  19 (35.8)  0  0  2 (3.8) | 0.131 |
| HVPG (mmHg), n=66 | 14.5  (10.0-19.0), n=40 | 16.0  (10.8; 20.5), n=26 | 0.314 | 16.0  (10.0; 20.0), n=53 | 14.0  (9.0;18.0), n=13 | 0.424 | 7.0  (5.0;8.0) | 17.0  (13.0;21.0) | <0.001 |
| Noninvasive biomarkers  sAxl (ng/ml)  sAxl/alb*10, n=380  Gas 6 (ng/ml)  Gas6/alb*10, n=380 | 61.41 (47.53;80.05)  15.16 (11.57;20.56)  56.78 (41.19;76.52)  14.09 (9.97;19.56) | 91.18 (73.25;119.34)  28.18 (21.78;40.72)  101.12 (77.89;122.65)  31.38 (22.10;41.78) | <0.001  <0.001  <0.001  <0.001 | 69.92 (52.35;86.81)  18.21 (13.23;25.42)  63.77 (47.02;90.09)  16.89 (11.30;25.0) | 104.24 (77.34;124.46)  31.68 (24.48;43.94)  109.51 (87.49;140.88)  33.77 (25.68;47.83) | <0.001  <0.001  <0.001  <0.001 | 51.66 (41.91;54.29)  11.80 (10.56;15.86)  41.16 (27.54;53.60)  10.31 (6.38;11.90) | 65.12 (46.84;77.80)  18.23 (11.93;25.47)  59.22 (43.68;81.337)  15.63 (10.91;25.82) | 0.086  0.039  0.015  0.004 |

**Supplementary Table S5. Patients with liver cirrhosis were compared with respect to CPS, MELD and HVPG.** CPS, Child Pugh Score; MELD, Model of End-stage Liver Disease; HVPG, hepatovenous pressure gradient; BMI, body mass, index; NAFLD, non-alcoholic fatty liver disease; ALD, alcohol-related liver disease; AIH, autoimmune hepatitis; PSC, primary sclerosing cholangitis; PBC, primary biliary cholangitis; VCTE, vibration controlled elastography.
