# Supplementary material for: Gas6 in chronic liver disease—a novel blood-based biomarker for liver fibrosis
Source: Cell Death Discov. 2023 Aug 2;9:282. doi: 10.1038/s41420-023-01551-6 (PMC10397215; doi:10.1038/s41420-023-01551-6)
Supplement: Supplementary file 8 — Supplementary Material legends [file 41420_2023_1551_MOESM8_ESM.docx]

**Supplementary Material**

**Supplementary Fig. 1**

**sAxl and Gas6 are associated with transplant-free survival in patients with liver cirrhosis.** Long-term follow-up was available in 256 patients. Transplant-free survival was calculated using the Kaplan-Meier method and stratified according to sAxl/alb and Gas6/alb levels. sAxl/alb and Gas6/alb cut-offs were identified based on Youden-Index, and sensitivity and specificity were calculated. Transplant-free survival was compared using the Log-rank test. Liver transplant and death were censored. **(A)** Patients were stratified by a sAxl/alb cut-off of 22.96 mg/dL (Youden Index = 0.342; n= 128 patients per group). Sensitivity and specificity were 64.8%, and 96.4%, respectively. Transplant-free survival was significantly lower in patients with sAxl/alb ≥ 22.96 mg/dL (p<0.001). **(B)** Patients were stratified by a Gas6/alb cut-off of 20.16 mg/dL (Youden Index = 0.249, n = 100 vs. 156 patients). Sensitivity and specificity were 71.7%, and 53.2%, respectively. Transplant-free survival was significantly lower in patients with Gas6/alb ≥ 20.16 mg/dL (p<0.001).

**Supplementary Fig. S2**

**sAxl and Gas6 in HCC patients.** **(A)** sAxl and Gas6 serum levels in HCC patients (n = 323) compared to CCA (n = 36) and CRCLM patients (n = 54). **(B)** Analysis of sAxl and Gas6 serum levels in HCC patients with cirrhosis (n = 267) and HCC patients without cirrhosis (n = 56) and healthy controls (n = 57). **(C)** Analysis of sAxl and Gas6 serum levels in HCC patients according to early BCLC stage (BCLC 0 + BCLC A, n = 128) and later BCLC stages (BCLC B, n = 81; BCLC C, n = 47, BCLC D, n = 15). Outliers are marked. HCC, hepatocellular carcinoma; CCA, cholangiocarcinoma; CRCLM, colorectal carcinoma liver metastases; BCLC staging, Barcelona Clinic Liver Cancer staging. n.s., not significant. Statistical significant differences are expressed as asterisks: ***P<0.001.
